# Racing Performance of the Quarter Horse: Genetic Parameters, Trends and Correlation for Earnings, Best Time and Time Class

**DOI:** 10.3390/ani13122019

**Published:** 2023-06-17

**Authors:** Ricardo Faria, António Vicente, Josineudson Silva

**Affiliations:** 1 Faculdade de Ciências Agrárias e Veterinárias, Universidade Estadual Paulista (Unesp), Jaboticabal 14884-900, SP, Brazil; 2 Hi-Tech Equine (HT Equine, Unipessoal LDA), 7330-313 Marvão, Portugal; 3 Escola Superior Agrária de Santarém (ESAS), Instituto Politécnico de Santarém, 2001-904 Santarém, Portugal; 4 CIISA—Centre for Interdisciplinary Research in Animal Health, Faculty of Veterinary Medicine, University of Lisbon, 1300-477 Lisboa, Portugal; 5 AL4AnimalS—Associate Laboratory for Animal and Veterinary Sciences, 1300-477 Lisboa, Portugal; 6 CIEQV—Life Quality Research Centre, Instituto Politécnico de Santarém, 2001-904 Santarém, Portugal; 7 Faculdade de Medicina Veterinária e Zootecnia, Universidade Estadual Paulista (Unesp), Botucatu 18618-307, SP, Brazil; 8 National Council for Science and Technological Development, Brasilia 71605-001, DF, Brazil

**Keywords:** equine, heritability, racecourse, racehorse, speed

## Abstract

**Simple Summary:**

The main goal in selecting racehorses for breeding is usually monetary earnings, and animals that earn the highest amounts of money are usually selected for breeding. These animals are not always the fastest on the racecourse because there are several competitions where the animals were faster but the prize money for the winners was lower. This practical strategy meant that the fastest racehorses were not always selected for breeding. The present study evaluated several traits through the results of heritability, genetic correlations and trends. Results from this study indicate that when we evaluated the earnings trait together with other important traits, the best values were observed. The selection process for the best sires and dams should be performed through two stages: first, by evaluating the animals that obtained the best times and, in the second stage, by evaluating the animals that had the highest monetary earnings. The design of breeding programs using the earnings trait in conjunction with another racing performance trait can change the results observed and improve genetic gains for speed racing Quarter Horses around the world.

**Abstract:**

The aim of this study was to evaluate the sprint racing performance of Quarter Horses in Brazil. Estimating genetic parameters, trends and correlations were obtained by single- and two-trait analyses using Bayesian inference (earnings to 2 years of equestrian age, best time and time class at distances of 301 m and 402 m). The data comprised a period of 38 equestrian years (1978 to 2015) with 23,482 sprint race records from 5861 animals. The heritability estimates were of low to moderate magnitude, ranging from 0.10 to 0.37 (single-trait) and from 0.15 to 0.41 (two-traits), and the repeatability was 0.31 to 0.46. The additive, residual and phenotypic correlations between earnings to 2 years of equestrian age and the other traits (best time and time class in distances 301 m and 402 m) were high (−0.95, −0.96, 0.69 and 0.92), low (−0.29, −0.37, 0.26 and 0.27) and moderate (−0.41, −0.47, 0.37 and 0.47), respectively. There is a positive genetic trend for all traits considered. However, evaluation of the last 10 equestrian years (2006 to 2015) showed negative trends (genetic loss) and trends close to zero (genetic stagnation). The design of breeding programs using the earnings trait in conjunction with other racing performance traits can enhance changes in the genetic gains as a whole in speed-racing Quarter Horses. These findings suggest that the traits studied should be included in breeding selection programs for racing Quarter Horses.

## 1. Introduction

The genetic origin of the Quarter Horse (QH) breed dates back to the 17th century, when English mares were bred with stallions from the regions of present-day Saudi Arabia and Turkey. The QH has become one of the largest and multifunctional horse populations in the world [1]. The use of QHs in family outings, working with cattle, Western competitions and sprint races has been preserved since the formation of the breed (17th century) until the 21st century. The official start of modern sprint QH races occurred in 1943 in the United States (Tucson, Arizona). In Brazil, information about the beginning of QH races is lacking, but zootechnical records date back to July 1978 (Sorocaba, São Paulo).

In evaluations of the racing performance of QHs in several studies, different methods have been used concerning the phenotypic traits of finish time [2], such as the ANOVA method and the effects of age and sex, with a range of 0.00 to 0.38 for heritability (h^2^). For the traits of time and final rank [3], the animal model by REML was used and included random animal and permanent environmental effects with racing, sex, age and origin as fixed effects; the resulting h^2^ ranged from 0.13 to 0.17. For race time and speed index [4], estimates were obtained by the MTGSAM program using animal models including the random additive genetic effect, random permanent environmental effect and the fixed effects of sex, age and race, with h^2^ ranging from 0.14 to 0.41. Earnings [5] were estimated by Bayesian inference (GIBBS2F90), and included the effects of sex, year of racing and animal in all analyses, with h^2^ ranging from 0.19 to 0.21. For the time class [6], the analyses used the same method as the previous study [5], and h^2^ values ranged from 0.45 to 0.56. On the other side, evaluating the racing performance of the most well-known breed of horse (Thoroughbred), many phenotypic traits for a single purpose (racing performance) have been presented over the last several decades (1950 to 2010). In the review [7], 15 phenotypic traits for racing performance were presented and distributed in three categories, time and its several variations (final, best and average time), handicap or similar performance ratings (categorical traits), earnings (annual, star, average and log earnings) and morphology (standard and measures).

Genetic evaluations for racehorses based on three different traits (money earnings, time and score) using the same database and animal model, to our knowledge, have not yet been observed in the literature. The possibility of evaluating three traits of different categories (earnings, time and time class as categorical traits) with the same database will provide more complete information, with discriminatory analysis. It also allows the genetic evolution of traits over the years and the relationship between the earnings trait and other traits to be verified. This is especially important for the earnings trait (i.e., money earned by the horse for its owner) since it is considered the most important trait.

The use of earnings as a trait in horses was first reported in 1948 [8], who called it the Average Earnings Index. This trait was altered in 1977 [9] and named the Standard Starts Index. The trait was later denominated as earnings in 1980 [10]. In the QH breed, earnings showed moderate heritability estimates (0.19 to 0.28) and positive genetic trends [5], suggesting its inclusion in selection programs. The phenotype of final time is widely used in the genetic evaluations of racehorses [7]. Within this context, the best time trait, which represents the fastest time of all races of a given animal, is the trait most used as a selection criterion [11]. The categorical trait time class unites animals in categories based on the time obtained by the winner of the race. Thus, the minimal differences between competitors are disregarded, suggesting that they are of environmental and non-genetic origin. For the time class trait in QH [6], high heritability and repeatability estimates were reported, suggesting the application of this trait in selection programs. The authors suggested that these animals be evaluated at a younger age and separately for each distance.

The aim of this study was to evaluate the sprint racing performance of Quarter Horses in Brazil by single- and two-trait analyses, estimating genetic parameters and trends for earnings at 2 years of equestrian age (E2), best time (BT) and time class (TC) at distances of 301 m (BT301 and TC301) and 402 m (BT402 and TC402), using the same database in the evaluation of the different traits.

## 2. Materials and Methods

### 2.1. Data Description

The sprint race records of QHs were provided by the Sorocaba Jockey Club and contained information for the period from July 1978 to June 2016. The races occurred in five racecourses in the cities of Sorocaba, São Paulo, Ribeirão Preto, Jaú and Avaré, all of them in the state of São Paulo, Brazil. There were 23,482 records from 5861 animals (42.2% males) compiled from 5138 races. The records corresponded to distances of 275, 301, 320, 365 and 402 m (1072; 6579; 2726; 5682 and 7423 records, respectively). The data analyzed account for a considerable proportion (single measures 57.8% of the total animals and repeated measures 55.2% of total records) of QH racehorses in Brazil (July 1978 to June 2016). For a better interpretation of the data, graphs were created showing the distribution of records by trait (Supplementary Figure S1a,b).

The equestrian age class of the horse is determined by the equestrian year regardless the date of birth. In the southern hemisphere, the equestrian year starts on July 1 and lasts until June 30 of the following year. In the present study, equestrian age classes of 2, 3, 4 or more years were used. To evaluate all QH animals with performance in each of the traits, the results of the genetic values and trends were analyzed separately by sex, breeding animals and equestrian ages. In the groups of horses evaluated (Table 1), 42.2% were males and, of these, 154 are breeding stallions (parents of 1676 athlete horses) with an average of 10.2 progenies; 57.8% were females, of which 964 are brood mares (mothers of 2811 athlete horses) with an average of 2.9 progenies, evaluating a total of 1118 breeders.

### 2.2. Description of the Traits and Data Files

#### 2.2.1. Earnings

Earnings represent the sum of monetary prizes earned by a horse over its lifetime career or at a specific age or period. In the present study, considering that 74.9% of the horses participated in their first race at 2 years of equestrian age and following the recommendations by Silva et al. [5], earnings at 2 years of equestrian age (E2) was defined as the trait. The prize money won at 2 years of equestrian age was obtained at different times (from 1978 to 2015) and in different Brazilian currencies [12]. The values obtained [5] were then converted to international currency (USD), and the monetary update was obtained for June 2019 [13]. Since the phenotypic values of E2 showed no normal distribution, log10 transformation was applied [14]. For the analysis of E2, only the records of races that had at least 1 or more monetary prizes were considered [15], resulting in a data file of 1900 animals, whose total prize moneys were summed when the animals had competed at the age of 2 equestrian years (Table 1).

The justification for evaluating E2 (2 years of age) was the possibility of selecting animals for breeding at the beginning of their performance career, the large number of records at 2 years of equestrian age available and the conclusions of other studies and authors [5,16]. The monetary prizes of the 1900 animals (E2) were related to 2963 classifications obtained at 2 years of age (37.8% of the total monetary prizes distributed between the years of 1978 and 2015 in Brazil). The monetary prize distributions of the 2963 classifications were obtained from the 1st to the 8th place, distributed as follows: 1st place (1054 monetary prizes), 2nd place (956 monetary prizes) and 3rd place (822 monetary prizes), and the 4th, 5th, 6th, 7th and 8th places have a collective total of 131 monetary prizes.

#### 2.2.2. Best Time

The best time (BT) of each animal was defined as the fastest final time of all races at a given distance in which the animal competed. In the present study, the distances of 301 m (BT301) and 402 m (BT402) were evaluated, with 3365 and 3318 individual records (animals), respectively (Table 1). The final times were recorded by automatic electronic timing in seconds.

#### 2.2.3. Time Class

The time class (TC) trait was divided into five different classes based on the final times (in seconds) of each race [6]. The scores for the formation of the classes were attributed within each race based on the percent difference in relation to the winner’s time. Class 5 includes animals with a final time <0.10% in relation to the winner’s final time, including the winner. Classes 4, 3, 2 and 1 include animals with a final time of 0.11% to 1.0%, 1.01% to 3.0%, 3.01% to 5.0% and >5.0% in relation to the winner’s final time, respectively. The phenotypic values of TC are repeated measures of the animals and were evaluated at distances of 301 m (TC301), with 6579 records, and 402 m (TC402), with 7423 records (Table 1).

The distance of 301 m (BT301 and TC301) was chosen because it provided the largest number of records for the 21st century and for the first race of the animals. The distance of 402 m (BT402 and TC402), corresponding to a quarter of a mile, was chosen because of its importance for the breed and because it had the largest number of total records. The selection of the traits is based on the importance they have, as determined in other studies [5,6,7,16].

### 2.3. Analyses

Single-trait analyses were performed to obtain the variance components and to explore the results compared to two-trait analysis. The latter was carried out considering E2 as the anchor trait and changing the other traits one by one, i.e., computing E2 with BT301 (3967 animals), E2 with BT402 (3946 animals), E2 with TC301 (7695 records) and E2 with TC402 (9088 records).

The model used for E2 included the fixed effects of equestrian year of birth, sex and number of starts as a covariate [10], in addition to additive genetic and residual effects. For BT301 and BT402, the model included the fixed effects of equestrian year of birth, sex, equestrian age class and racecourse, as well as additive genetic and residual effects. For TC301 and TC402, the model included the systematic effects of sex, equestrian age and racecourse, and the random effects of animal, the permanent environment effects of the animal and residual effects.

The pedigree file used in all analyses contained 11,425 animals. The quality of the genealogical information was 5.5 ± 1.2 known equivalent generations [17], with 1855 sires and 4858 dams.

All models used for the evaluation of E2, BT301 and BT402 (linear) and of TC301 and TC402 (threshold) can be written in matrix form as:

$$y=X\beta +Z_{1}\alpha +Z_{2}c+e$$

where **y** is the vector of observations; **β** is the vector of fixed effects; **α** is the vector of the direct additive genetic effects of the animal; **c** is the vector of permanent environmental effects of the animal (only for TC301 and TC402); **e** is the vector of random residual effects, and **X**, **Z_1_** and **Z_2_** are incidence matrices that relate the observations to the fixed effects, random direct additive genetic effects and uncorrelated permanent environmental effects, respectively.

For the threshold model (TC301 and TC402), it was assumed that the underlying scale shows a continuous normal distribution described as:

$$U|\theta \sim N\left(W\theta ,Ι\sigma_{e}^{2}\right)$$

where **U** is the vector of the underlying scale of order **r**; **Ɵ**’ = (**β**’, **α**’, **c**’) is the vector of the location parameters of order **s**, with **β** being defined as systematic effects, **α** as additive genetic effect and **c** as the permanent environmental effect of the animal; **W** is the known incidence matrix of order **r** by **s**; **I** is the identity matrix of order **r** by **r**; and $\sigma_{e}^{2}$ is the residual variance.

In the analysis of categorical variables, vectors **β**, **α** and **c** are location parameters with a conditional distribution **y**|**β**, **α**, **c**. A uniform prior distribution was assumed for **β**, which reflects vague prior knowledge about this vector. Inverse Wishart distributions were attributed to the remaining components. Thus, the distribution of **y**, given the scale parameters, was assumed to be:

$$\left(y|\beta ,\alpha ,c,R\right)\sim N\left[Χ\beta +{Ζ}_{\alpha }\alpha +W_{c}c,{Ι}_{N}R\right]$$

TC301 and TC402 are categorical traits that are determined by unobservable continuous variables on an underlying scale, in which the initial threshold values are fixed: **t1 < t2** ... < **tj − 1**, with **t0** = **− ∞** and **tj** = **∞**, where j is the number of categories. The categories or scores of **yi** (TC trait) for each animal **i** are defined by **Ui** on the underlying scale:

*Yi = (1) t0 < Ui ≤ t1; (2) t1 < Ui ≤ t2; (3) t2 < Ui ≤ t3; (4) t3 < Ui ≤ t4; (5) t4 < Ui ≤ t5, for i = 1, ..., n,*

where **n** is the number of observations. After specification of the thresholds **t0** to **t5**, 1 of the thresholds (**t0** to **t5**) needed to be adjusted to an arbitrary constant. In this study, **t1** = **0** was considered, with the vector of estimable thresholds being defined as **t** = **t2**, **t3** and **t4**.

According to the Bayesian approach, in the two-trait analyses involving continuous (E2) and categorical (TC) variables, the initial distributions of the random genetic, uncorrelated and residual effects were assumed to follow a multivariate normal distribution as follows:

$$\begin{array}{c} p([\begin{array}{c} a_{1}\\ a_{2} \end{array}]|G)\sim Ν\left(\left[\begin{array}{c} 0\\ 0 \end{array}\right],G=G_{0}⊗A\right),p(\begin{array}{c} c_{1}\\ c_{2} \end{array}|C)\sim Ν\left(\left[\begin{array}{c} 0\\ 0 \end{array}\right],C=C_{0}⊗I\right),\\ p(\begin{array}{c} e_{1}\\ e_{2} \end{array}|R)\sim Ν\left(\left[\begin{array}{c} 0\\ 0 \end{array}\right],R=R_{0}⊗I\right) \end{array}$$

where **G0** is the genetic variance and covariance matrix; **C0** is the variance matrix of uncorrelated effects; **R0** is the residual variance matrix; **⊗** is the direct product operator; **A** is the relationship matrix; and **I** is the identity matrix.

Initial distributions of the (co)variances were assumed as inverse Wishart distributions for random genetic, uncorrelated and residual effects of the traits studied, including the covariance between them. Uniform prior distributions were defined for the fixed effects and thresholds. The degree of freedom corresponding to the inverse Wishart distribution, which indicates the level of reliability of the initial distribution (v), was flat for all initial variances, i.e., it did not reflect the degree of knowledge about the parameters (v = 0) [18].

For single-trait analysis under linear and threshold animal models and two-trait analysis under linear–linear and linear–threshold models, Gibbs chains of 1,600,000 cycles for E2 and of 1,100,000 cycles for the other traits were run, with burn-in periods of 600,000 and 100,000 cycles, respectively. These numbers were chosen after verification of the stationary stage of the chain by graphical inspection [19]. The estimates were stored every 20 samples, totaling 50,000 samples. These samples were used to compute the posterior means, standard deviations and 95% highest posterior density intervals (HPD95) of the variance components, heritability, repeatability and genetic and residual correlations. The statistical analyses were performed with the GIBBSF90 test and THRGIBBS1F90 programs [20]. The latter was used for analysis of the categorical traits TC301 and TC402.

The results of single- and two-trait analyses were evaluated regarding convergence of the chain using the POSTGIBBSF90 program [20]. The established chain lengths (1,100,000 and 1,600,000 cycles) and burn-in periods (100,000 and 600,000 cycles) were sufficient to obtain convergence in the analyses [20]. The minimum effective sample size (ESS) of the (co)variance components indicated the number of independent samples with information equivalent to that present in the dependent sample [21]. The ESS estimates obtained in the present study were higher than 200 cycles, indicating convergence of the Gibbs chain.

The posterior estimated breeding values (EBV) were obtained by BLUP procedures using OPTION fixed_var mean [20] from the genetic and residual (co)variances obtained in the final analysis of the present study. The description of the EBV for the 5 traits corresponded to 1900 (E2), 3365 (BT301 and TC301) and 3318 (BT402 and TC402) animals born between 1971 and 2014, a period comprising the first and last births of animals with racing records in Brazil, respectively. The EBV for breeding stallions and broodmares over the same period are also described. A t-test was used to compare means between groups.

Genetic trends were calculated by linear regression of EBVs on year of birth of the animals (from 1971 to 2014). The results are presented in the form of graphs of the annual means for the five traits and their respective linear trend lines.

## 3. Results

### 3.1. Heritability (h^2^) and Repeatability

The values of the additive genetic variation components $(\sigma_{g}^{2}$) were higher in all two-trait analyses than the one-trait analyses (Table 2). The values of the residual variance ($\sigma_{e}^{2}$) followed the same trend of $\sigma_{g}^{2}$, with the exception of the two-trait analysis between E2 × TC301 and E2 × TC402; in addition, the values of $\sigma_{e}^{2}$ were higher than the values $\sigma_{g}^{2}$ (Table 2), the difference being more than double the $\sigma_{e}^{2}$ values in uni-trait analyses (E2, BT301 and TC301) and two-trait analyses (E2 × BT301, E2 × BT402 and E2 × TC301, BT301 × E2 and TC301 × E2). At longer distances (BT402 and TC402), smaller differences were observed between the values of $\sigma_{g}^{2}$ and $\sigma_{e}^{2}$ (Table 2). The estimates of the permanent environmental variance ($\sigma_{c}^{2}$) showed lower values than $\sigma_{g}^{2}$ and $\sigma_{e}^{2}$.

The heritability (h^2^) estimates obtained in the single-trait analyses were low for E2, BT301 and TC301 and moderate for BT402 and TC402 (Table 2). The shortest HPD95 intervals were observed for BT301 and BT402 and the highest interval was observed for TC402 (Table 2). The repeatability estimates for the two TC traits were moderate.

The h^2^ estimates obtained in the two-trait analyses were higher than those of single-trait analysis (Table 2). The h^2^ estimates continued to be low for the E2 when evaluated with the distance of 301 m (E2 × BT301 and E2 × TC301) and moderate at the distance of 402 m (E2 × BT402 and E2 × TC402). Moderate h^2^ estimates were obtained for the distance of 301 m (TC301 × E2 and BT301 × E2) and high estimates for the longest distance of 402 m (BT402 × E2 and TC402 × E2). The central measures showed symmetry in the single- and two-trait analyses, and the standard deviations showed small magnitudes, except for the TC402 trait (Table 2).

The HPD95 intervals for the h^2^ estimates exhibited low to moderate variation in the two-trait analyses. The highest interval was observed for E2 × TC402 (Table 2). The repeatability estimates for the two TC traits were higher than those obtained in single-trait analysis.

### 3.2. Genetic Correlations

The lowest and highest values of r_g_ and r_p_ were observed for the evaluation of E2 × TC301 and E2 × BT402, respectively. The lowest estimates of r_r_ were observed for E2 × BT301, the highest values of r_r_ were similar or equal for the other traits (Table 3). The negative correlations between E2 and BT indicate that when E2 increases (greater earnings), BT decreases (racing faster or completing races in a shorter time).

### 3.3. Estimated Breeding Values and Genetic Trends for Uni-Trait Analyses

The average EBVs above zero for traits E2, TC301 and TC402 and below zero (negative) for BT301 and BT402 are considered, in both scenarios, positive EBVs, given that the lower the value the better for the BT trait. Comparisons of EBV should be made within each trait and not as a comparison between traits.

The mean EBV between males and females (sex) indicated significant differences for the evaluated traits, except for TC301, and males had higher EBVs than females in all traits (Table 4). Between non-breeding and breeding animals, the EBVs of non-breeding animals were significantly higher (*p*-value < 0.05) for traits BT301 and BT402, higher in TC301 (not significant, *p*-value > 0.05), equal in the E2 trait and significantly lower than breeding animals in TC402 (*p*-value < 0.05). Among sires and dams (stallions and mares), the EBVs were significantly different and higher in stallions for E2 and TC402 and in mares for BT301 and BT402, with higher EBVs also being seen in mares for TC301, but the differences were not significant. The EBVs within each trait regarding equestrian age (for E2; all other ages were obtained from pedigree relationship) decreased with advancing age for all traits, indicating the highest and lowest EBVs were observed for animals at 2 and 4 years of equestrian age, respectively. With the exception of TC301, EBV differences between all ages were significantly different (Table 4).

**Figure 1:**
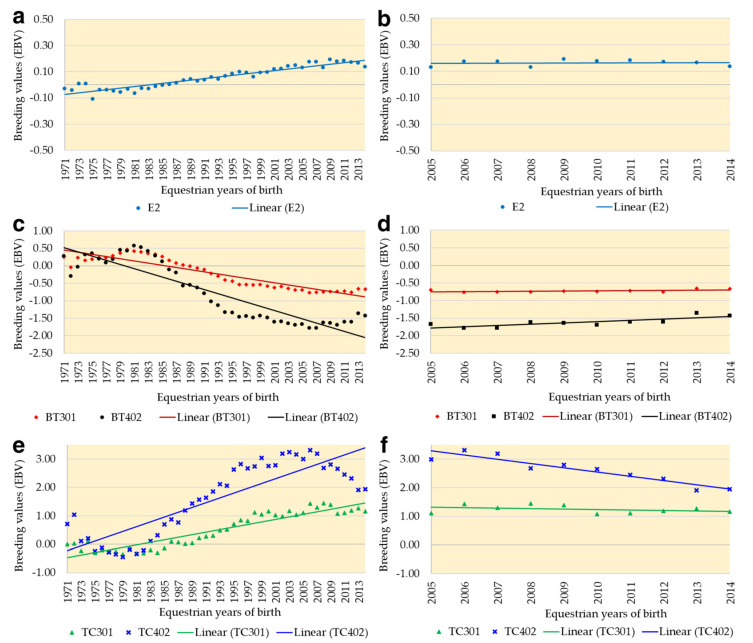
Linear genetic trends over 44 equestrian years of birth (1971 to 2014) and the last 10 years (2005 to 2014) for univariate traits Earnings at 2 years (E2) in (**a**,**b**); Best time at 301 m (BT301) and 402 m (BT402) in (**c**,**d**); and Time class at 301 m (TC301) and 402 m (TC402) in (**e**,**f**) obtained for racehorses of the Quarter Horse breed in Brazil.

The genetic trends over 44 years (1971 to 2014) obtained by univariate trait analyses and according to year of birth of the animals is presented in Figure 1. The negative genetic trends for BT301 and BT402 are favorable (Figure 1a) since lower values of BT are desirable. Oscillations were observed in animals born in the first 10 years (until 1980), followed by considerable improvements until the beginning of the 21st century (Figure 1a,c,e). Analysis of the last 10 years of birth (Figure 1b,d,f) showed values opposite to all equestrian years evaluated, indicating an annual decrease in the EBVs of the racing population of the QH breed in Brazil.

## 4. Discussion

The h^2^ estimates of the present study, obtained through single-trait analysis (Table 2), are close to those reported in studies evaluating the performance of QHs. The earnings trait obtained an h^2^ of 0.16 to 0.28 [5]. For final time, h^2^ estimates of 0.20 to 0.38 [2,22], of 0.26 to 0.41 [4], and of 0.17 [3] were reported. Using multi-trait analysis [6], an h^2^ of 0.45 to 0.56 was estimated for TC. Earnings, a trait used to evaluate the performance of Thoroughbred racehorses in Italy [23], France [24], Germany [25] and the Czech Republic [26], had h^2^ estimates of 0.12 to 0.13, 0.02 to 0.06 and 0.10 to 0.19, respectively, which are equally low as those obtained in the present study (Table 2).

The higher h^2^ estimates obtained in the two-trait analyses of BT402 × E2 and TC402 × E2 (Table 2) suggest that a combined evaluation provides more genetic information. The bivariate models are theoretically more accurate given that they consider the information on both traits to simultaneously estimate the random effects [27].

Evaluating BT in Thoroughbred racehorses obtained high (0.58 to 0.77), moderate (0.32 to 0.40) and low (0.10 to 0.23) h^2^ estimates for 2 years, 3 years and considering all ages, respectively [28]. In Brazil, a low h^2^ was reported (0.12) for BT in Thoroughbred animals [29]. Using earnings and BT data of Arabian racehorses also yielded low (0.14 to 0.17) and moderate (0.23 to 0.36) h^2^ estimates, respectively [30].

The BT and E2 traits use racing performance, but with different definitions, which we can consider to be complementary to each other; i.e., it does not matter if a horse is the fastest without making money (qualifying) or if it earns a lot of money without being the fastest. By evaluating in model bivariate, they complement each other and allow for the obtaining of major h^2^ values (Table 2). in addition to making the genetic values available to breeders for both traits and considered the value of genetic correlation (Table 3).

The h^2^ estimates reported in the literature and those of the present study (Table 2) were similar, suggesting that the differences were possibly due to the different analysis methods, models and populations used in each study. The higher standard deviations in the analysis of the TC402 trait are possibly due to the greater variability in the competitors in the 402 m race, which has the highest participation; that is, due to the visibility of this event, owners strive to ensure that their animals win these races through the best training, nutrition and veterinary care. At the same time, however, there are breeders who do not carry out such preparation for the competition resulting in horses who finish with greater time differences from the winners. Thus, placing animals without the capacity to win in the same races as highly competitive horses results in greater variability when we evaluate data from the longest distance races (402 m). Among the characteristics evaluated, those with higher h^2^ values (Table 2) indicate a greater possibility of genetic gains over the generations, suggesting their use as a selection criterion in the breeding programs of speed racing horses.

The repeatability estimates reported for QH in Brazil evaluating the distances of 301 and 402 m were 0.28 and 0.42, respectively, for the speed index, and 0.36 and 0.68, respectively, for final time [4]. In the United States, Mexico and Canada, the repeatability estimates at the distance of 402 m were 0.32 for final time; the authors did not provide results for the distance of 301 m but reported a repeatability of 0.36 evaluating animals at 2 years of age [2]. The moderate repeatability estimates obtained in the present study (Table 2) and in the cited literature indicate that the capacity of the animal to repeat the phenotypic value at each distance was moderate. For the selection and culling of animals, the largest number of possible measures should be taken into consideration [4]. On the other hand, multi-trait analysis of TC at different distances and the evaluation of a large number of repeated measures per QH animal [6] obtained high repeatability estimates (0.78 to 0.97), ensuring greater accuracy at the time of selection and culling of racehorses when compared to the results of single- and two-trait analyses of TC in the present study. These results indicate a more robust analysis can be obtained when including repeated records of horses in different distances. Considering that only 101 m (average difference of 5 s) separates the shortest (301 m) and the longest (402 m) distance, it is normal for the same animal to compete in both distances. Thus, it is common for animals, progenies and ancestors of QHs to have records at different distances.

Correlation estimates allow the behavior of a trait to be evaluated when selection is performed for another trait, and thus, to obtain genetic gain (indirect selection) in one trait through another that is difficult to measure or whose heritability is low [31]. The high genetic correlations (r_g_) observed in the present study (Table 3) were also observed in the literature [3,4,5] evaluating the racing performance of QHs. The high r_g_ estimates suggest that selection for E2 can have benefits for BT at both distances and for TC at the longer distance. Considering the direct selection for BT and TC traits, this is the most efficient way to obtain improvement in these traits. Furthermore, joint selection with the E2 characteristic, in addition to increasing the values of both BT and TC, allows the selection of animals early.

The magnitudes of the residual correlations (Table 3) suggest that the same environmental factors affect the evaluated traits in different ways. The phenotypic correlations (r_p_) followed the trends of r_g_ (Table 3), but the estimates were lower. The results obtained indicate that genetic factors exert a greater influence than environmental factors in QH racehorses in Brazil.

The performance of an elite horse is the sum of its genetic capacity (transmitted by its ancestors) and environmental influences (management, nutrition, training, horseman and other random effects). The EBVs are estimates of the animal’s genetic capacity and can be used for the calculation of sports performance [6], morphology [32], health [33] and for the selection and culling of breeding animals.

The EBVs obtained (Table 4) indicate variability in the QH population, confirmed by the standard deviations. Faria et al. [6] suggested that QHs with superior EBVs would not be selected for breeding in view of the lower EBVs of breeding stallions compared to the average of the QH population. In the present study, this statement can be confirmed for breeding stallions by BT301, BT402 and TC301; for the remaining traits (E2 and BT402), EBVs were higher for males. Differences presented in Table 4 indicate that breeding females, compared to stallions, have genetic equality or superiority, possibly given the higher number of offspring per stallion, diluting their genetic gain. In the case of brood mares, the evaluation of EBVs for single traits (E2, BT301 and BT402) indicated that the best females are not being selected as breeding animals. However, analysis of the repeated measures (TC301 and TC402) showed that females with higher EBVs produce offspring in the population, i.e., they are selected as breeding animals. The results obtained for TC in the present study indicate that it is a valuable trait for the assessment and selection of elite QH racehorses for breeding.

The lack of selection of horses with superior EBVs for breeding impairs the evolution of racehorses of the QH breed, as observed in the present study by the EBV values between non-breeding and breeding animals (Table 4) and by the analysis of the genetic trends observed in the last 10 years (Figure 1). Breeding animals should be selected based on the correct and precise evaluations of horses, and breeders should request the help of researchers in the evaluation and selection of breeding animals, thus ensuring phenotypic improvements in QHs whose sprint race records have stagnated since 2009 [34].

There was a decrease in EBVs for all traits with advancing equestrian age (Table 4), suggesting that the animals can be selected early for breeding, i.e., immediately after completing 2 years of equestrian age. This approach makes it possible to reduce the long generational intervals (>10 years) of this population [12]; in fact, the generational interval is one of the limiting factors in the genetic improvement of horse breeding programs.

The genetic trends (Figure 1) indicated greater genetic progress in the longer distance races (BT402 and TC402). In North America [35], the authors reported opposite results, with greater progress at the shorter distance evaluated (320 m) and lower progress at 402 m. The difference between the shorter and longer distances in North America was explained by the large number of records at 320 m, suggesting that the training of the animals focused on this distance. In the present study, the same reason possibly explains the higher genetic trends at the distance of 402 m, which possessed a larger number of records (Table 1).

The genetic trends have decreased over the last 10 years (2005 to 2014) (Figure 1), a finding that suggests possible stagnation in the genetic evolution of the racing line of the QH breed in Brazil, comparable to that reported for the Thoroughbred breed [7]. The stagnation and genetic losses observed in the last decade (Figure 1) must be recovered through targeted mating, with an increase in the number of breeding animals with origins different from the ancestors of the QH breed in Brazil, as suggested in the populational study [36], who observed a genetic bottleneck in the current population of the racing line of the QH breed in Brazil.

## 5. Conclusions

Overall, this study emphasizes the lack of genetic response in recent years of the selection/evaluation of QHs bred in Brazil. The decline in genetic response was confirmed by the absence of significant genetic gains for the traits analyzed. The earnings trait exhibits genetic variability and genetic correlations favorable with a racing performance trait (best time and time class), suggesting that including the earnings trait in selection programs along with other racing performance traits may resulting in positive changes for the genetic trends. Quarter Horse associations, technicians and breeders must apply different strategies in selection and improvement programs that will allow for the genetic evolution of this population in conjunction with the monetary gains of the owners.

## Figures and Tables

**Table 1:** Descriptive statistics of the five racing traits and their respective phenotypic means in sprint racehorses of the Quarter Horse breed in Brazil.

Item	Trait						
	Single Measures				Repeated Measures		
	E2 ± sd (log10 $)	BT301 ± sd (s)	BT402 ± sd (s)	Total (No.)	TC301 ± sd (pt)	TC402 ± sd (pt)	Total (No.)
Mean trait	3.05 ± 0.91	17.33 ± 0.68	23.06 ± 1.13	-	3.63 ± 1.05	3.42 ± 1.18	-
No of animals ^a^ and records ^b^	1900 ^a^	3365 ^a^	3318 ^a^	5861	6579 ^b^	7423 ^b^	23,482
No of males ^a^ and records ^b^	860 ^a^	1436 ^a^	1438 ^a^	2474	2909 ^b^	3415 ^b^	10,335
No of females ^a^ and records ^b^	1040 ^a^	1929 ^a^	1880 ^a^	3387	3670 ^b^	4008 ^b^	13,127
No of stallions ^c^ and records ^d^	51 ^c^	92 ^c^	132 ^c^	154	296 ^d^	503 ^d^	1227
No of mares ^c^ and records ^d^	314 ^c^	538 ^c^	691 ^c^	964	1177 ^d^	1772 ^d^	4982

E2, earnings at 2 years of equestrian age; BT301 and BT402, best time at 301 and 402 m; TC301 and TC402, time class at 301 and 402 m; sd, standard deviation; log10 $, base 10 logarithm to dollars; s, seconds; pt, points; No., number; ^a^, total animals, males and females evaluated in single traits E2, BT301 and BT402 (single measures); ^b^, total records evaluated in repeated trait TC301 and TC402 (repeated measures) belonging to the animals evaluated in the BT301 and BT402 traits, respectively; ^c^, total breeding stallions and broodmares with racing records in Brazil and evaluated in single traits E2, BT301 and BT402 (single measures); ^d^, total records evaluated in repeated trait TC301 and TC402 (repeated measures) belonging to the breeding stallions and broodmares with racing records in Brazil and evaluated in the BT301 and BT402 traits, respectively.

**Table 2:** Posterior estimates of heritability and repeatability (including the additive genetic, residual and permanent environmental variation) in single- and two-trait analyses of E2, BT301, BT402, TC301 and TC40 in sprint racehorses of the Quarter Horse breed in Brazil.

Trait			Variation ( σ)			Heritability (h^2^)				ESS	Repeatability ± sd
			$\sigma_{g}^{2}$	$\sigma_{e}^{2}$	$\sigma_{c}^{2}$	Mean ± sd	Median	Mode	HPD95		
E2	Single		0.05	0.44	-	0.10 ± 0.04	0.10	0.10	0.02 to 0.19	516	-
	Two	BT301	0.08	0.44	-	0.16 ± 0.04	0.15	0.17	0.09 to 0.24	339	-
		BT402	0.06	0.22	-	0.22 ± 0.03	0.22	0.22	0.18 to 0.31	938	-
		TC301	0.07	0.41	-	0.15 ± 0.05	0.15	0.20	0.07 to 0.25	520	-
		TC402	0.18	0.36	-	0.33 ± 0.14	0.33	0.33	0.08 to 0.55	235	-
BT301	Single		0.03	0.10	-	0.23 ± 0.04	0.23	0.23	0.15 to 0.32	2500	-
	Two	E2	0.04	0.13	-	0.24 ± 0.04	0.24	0.23	0.16 to 0.32	549	-
BT402	Single		0.16	0.25	-	0.39 ± 0.04	0.39	0.35	0.30 to 0.43	6250	-
	Two	E2	0.19	0.27	-	0.41 ± 0.04	0.41	0.40	0.33 to 0.49	4232	-
TC301	Single		3.04	12.18	2.48	0.17 ± 0.06	0.17	0.14	0.08 to 0.32	280	0.31 ± 0.09
	Two	E2	3.58	13.24	2.52	0.19 ± 0.06	0.19	0.20	0.10 to 0.34	581	0.32 ± 0.08
TC402	Single		1.92	3.79	0.68	0.30 ± 0.13	0.30	0.33	0.25 to 0.43	360	0.43 ± 0.19
	Two	E2	3.55	5.99	1.01	0.34 ± 0.13	0.34	0.40	0.20 to 0.55	248	0.46 ± 0.17

$\sigma_{g}^{2}$, additive genetic variation; $\sigma_{e}^{2}$, residual variance; $\sigma_{c}^{2}$, permanent environmental variance; HPD95, 95% highest posterior density; ESS, effective sample size; E2, earnings at 2 years; BT301, best time at 301 m; BT402, best time at 402 m; TC301, time class at 301 m; TC402, time class at 402 m; sd, standard deviation.

**Table 3:** Genetic, residual and phenotypic correlations between E2 and BT301, BT402, TC301 and TC402 in sprint racehorses of the Quarter Horse breed in Brazil.

Trait	E2		
	r_g_ ± sd	r_r_ ± sd	r_p_ ± sd
BT301	−0.95 ± 0.07	−0.29 ± 0.04	−0.41 ± 0.03
BT402	−0.96 ± 0.03	−0.37 ± 0.04	−0.47 ± 0.03
TC301	0.69 ± 0.13	0.26 ± 0.05	0.37 ± 0.04
TC402	0.92 ± 0.08	0.27 ± 0.09	0.47 ± 0.07

E2, earnings at 2 years; BT301, best time at 301 m; BT402, best time at 402 m; TC301, time class at 301 m; TC402, time class at 402 m; r_g_, genetic correlation; r_r_, residual correlation; r_p_, phenotypic correlation; sd, standard deviation.

**Table 4:** Mean estimated breeding values of E2, BT301, BT402, TC301 and TC402 univariate traits for all animals and according to sex, breeding animal and equestrian age in racehorses of the Quarter Horse breed in Brazil.

Item		Trait Mean ± sd				
		E2 (log10 $)	BT301 (s)	BT402 (s)	TC301 (pt)	TC402 (pt)
Sex	Males	0.08 ± 0.20 ^a^	−0.34 ± 0.46 ^a^	−1.02 ± 0.90 ^a^	0.66 ± 1.23 ^a^	1.97 ± 1.67 ^a^
	Females	0.07 ± 0.19 ^b^	−0.26 ± 0.48 ^b^	−0.88 ± 0.91 ^b^	0.53 ± 1.23 ^a^	1.76 ± 1.69 ^b^
Nonbreeding		0.08 ± 0.20 ^a^	−0.32 ± 0.48 ^a^	−0.96 ± 0.91 ^a^	0.60 ± 1.22 ^a^	1.82 ± 1.66 ^b^
Breeding		0.08 ± 0.22 ^a^	−0.20 ± 0.47 ^b^	−0.83 ± 0.88 ^b^	0.58 ± 1.31 ^a^	1.96 ± 1.81 ^a^
BA	Stallions	0.08 ± 0.23 ^a^	−0.16 ± 0.52 ^b^	−0.76 ± 1.01 ^b^	0.56 ± 1.37 ^a^	1.99 ± 2.21 ^a^
	Mares	0.07 ± 0.20 ^b^	−0.20 ± 0.46 ^a^	−0.84 ± 0.85 ^a^	0.61 ± 1.29 ^a^	1.95 ± 1.73 ^b^
Age	2 years	0.08 * ± 0.21 ^a^	−0.32 ± 0.48 ^a^	−1.00 ± 0.90 ^a^	0.63 ± 1.23 ^a^	1.96 ± 1.68 ^a^
	3 years	0.06 * ± 0.16 ^b^	−0.23 ± 0.46 ^b^	−0.78 ± 0.90 ^b^	0.47 ± 1.20 ^b^	1.55 ± 1.68 _b_
	4 years	0.04 * ± 0.15 _c_	−0.13 ± 0.41 _c_	−0.55 ± 0.85 ^c^	0.32 ± 1.21 ^b^	1.26 ± 1.59 ^c^
	Total	0.08 ± 0.19	−0.29 ± 0.47	−0.94 ± 0.90	0.58 ± 1.23	1.85 ± 1.69

E2, earnings at 2 years; log10 $, base 10 logarithm to dollars; BT301, best time at 301 m; s, seconds; BT402, best time at 402 m; TC301, time class at 301 m; pt, points; TC402, time class at 402 m; sd, standard deviation; BA, breeding animal; different letters ^a^, ^b^ or ^c^ indicate significant differences *p*-value < 0.05; *, values obtained by the EBV of the pedigrees that started at age 3 years and 4 years or more.

## Data Availability

The data are not available for privacy reasons and belong to the Brazilian Association of Quarter Horse Breeders (ABQM) and Jockey Clube de Sorocaba (JCS) in Brazil.

## Supplementary Materials

The following supporting information can be downloaded at: https://www.mdpi.com/article/10.3390/ani13122019/s1, Figure S1: Distribution of data from Table 1 by traits E2, earnings at 2 years of equestrian age; BT301 and BT402, best time at 301 and 402 m; TC301 and TC402, time class at 301 and 402 m.
