# Supplementary figures and images for: Racing Performance of the Quarter Horse: Genetic Parameters, Trends and Correlation for Earnings, Best Time and Time Class
Source: Animals (Basel). 2023 Jun 17;13(12):2019. doi: 10.3390/ani13122019 (PMC10294889; doi:10.3390/ani13122019)

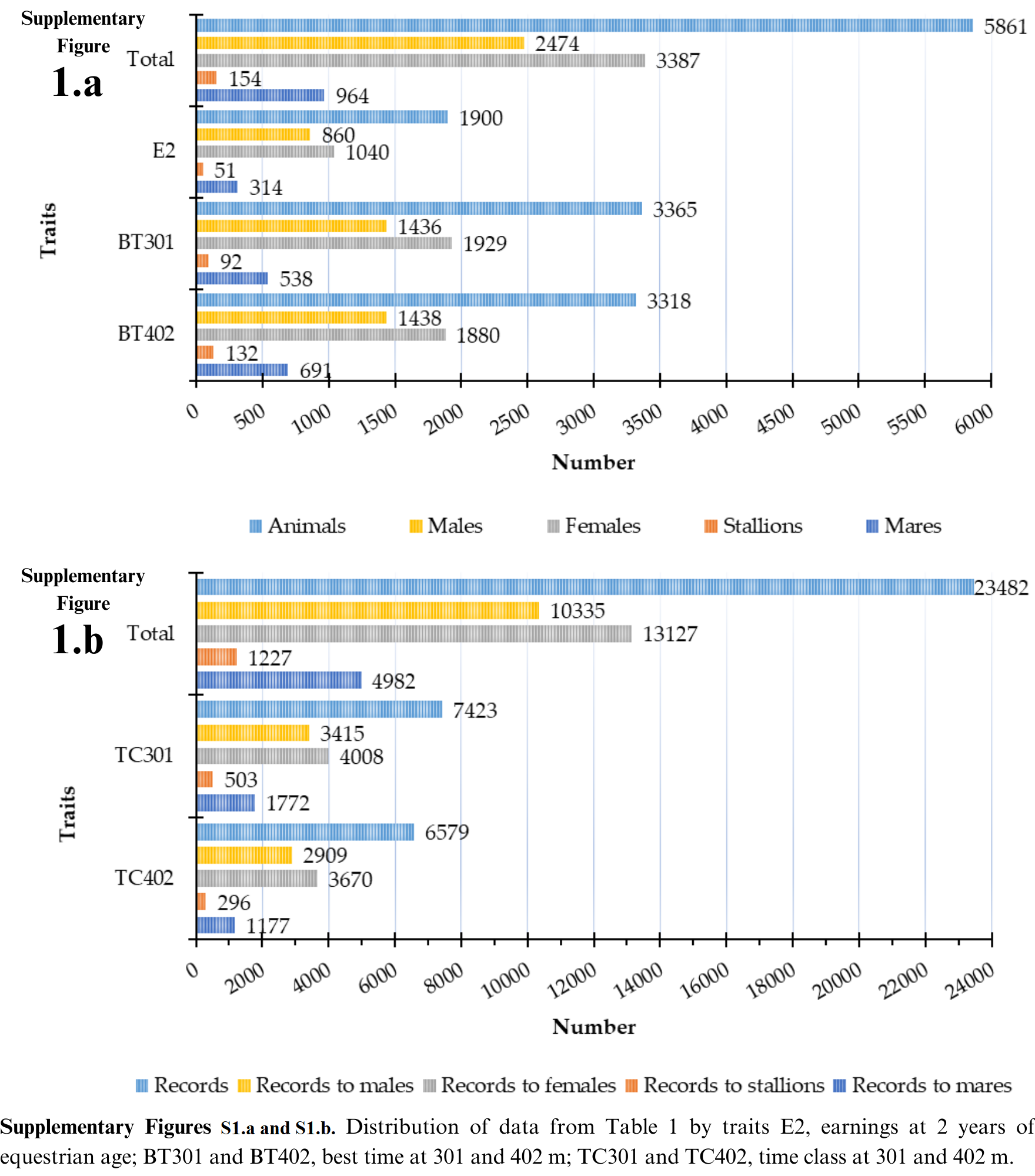

Supplement: Supplementary file 1 [file animals-13-02019-s001.zip › animals-2276801-supplementary.png]
